# Health symptoms and associated factors in times of a pandemic: a population-based study

**DOI:** 10.1186/s13690-024-01492-1

**Published:** 2025-01-13

**Authors:** J. Gerbecks, C. Plevier, C. J. Yzermans, M. L. A. Dückers, C. Baliatsas

**Affiliations:** 1 Netherlands Institute for Health Services Research (Nivel), Utrecht, The Netherlands; 2 Community Health Service Region Utrecht (GGD Utrecht), Utrecht, The Netherlands; 3 Otterstraat 118-124, Utrecht, 3513 CR The Netherlands

**Keywords:** COVID-19, Health symptoms, Non-specific symptoms, Pandemic

## Abstract

**Background:**

Non-specific symptoms, such as headaches and sleep problems, are more common after disasters. They can become chronic, and impact emotional and physical functioning. However, limited research has focused on such symptoms in the context of a pandemic. This study investigated the association between perceived impact of the COVID-19 pandemic, and prevalence, duration, and severity of health symptoms.

**Methods:**

A cross-sectional survey using validated questionnaires was conducted shortly after the first COVID-19 wave in 2020, with nearly 46,000 adult participants from Utrecht, the Netherlands. Negative binomial regression analyses were performed to assess the relationship between pandemic-related factors and symptom reports, adjusting for demographics, chronic conditions, lifestyle, and socio-economic status.

**Results:**

Perceived impact of the pandemic on stress levels, loneliness, anxiety and depression was consistently and significantly associated with symptom report, duration, and perceived severity. Incidence rate ratio’s (IRR) varied from 1.17 to 1.29. Delayed care during the pandemic was associated with severity of symptoms (IRR = 1.63; 99% confidence intervals (CI): 1.20–2.20). People that (suspected) got COVID-19 infected were at higher risk of symptom report, duration, and perceived severity (IRR around 1.20–1.28).

**Conclusion:**

As with other disasters, the perceived impact of an immediate threat such as a pandemic can influence health symptoms, independent of health or socio-demographic factors. Understanding symptom patterns and risk factors can assist healthcare professionals and policymakers in identifying vulnerable groups, symptoms profiles, and improving care and support during and after pandemics.

**Table:** 

Text box 1. Contributions to the literature
• Previous studies identified several symptoms (e.g. sleep quality, fatigue, anxiety, depression) that increased in prevalence during and remained after the pandemic;
• Most studies focused on prevalence of health problems related to infection with the virus;
• A large body of studies focused mainly on psychiatric symptoms in relation to the pandemic in general;
• This study adds to this literature by looking at perceived impact of the pandemic and its relation to prevalence, duration, and severity of symptoms with both physical and mental health symptoms;
• Providing a more complete image of health symptoms in times of the COVID-19 pandemic.

## Introduction

The prevalence of health symptoms such as headache, muscle pain, and sleep problems often increases after disasters, including pandemics. These symptoms are frequently classified as ‘non-specific symptoms’ (NSS), as their etiology can be unclear, unknown, or differ from patient’s attributions [1]. Fifty-six to ninety-one percent of the self-reported problems are classified as NSS by a general practitioner [2]. Reviews show NSS are common after disasters, and more prevalent in those affected by disasters compared to the general population [2–4].

The SARS-CoV-2 outbreak in December 2019 led to global spread of the virus and mitigation measures such as social distancing. These new public health measures and the perceived threat of the pandemic led to secondary physical and mental health issues [5–7], and likely higher levels of stress, since social connections help people regulate emotions, cope with stress and remain resilient during difficult times [8]. A recent review of COVID-19 literature indicated increased mental health problems, especially depression, increased during the pandemic and social restrictions [7]. According to a large body of international studies, the COVID-19 crisis had an impact on physical, mental and social health and appeared to worsen already existing psychological or physical problems [5, 6]. COVID-19 survivors for example still experienced poor sleep quality, fatigue, anxiety, depression and PTSD 1 year after infection, with a larger mental impact on the individuals with lower educational levels [9]. Witteveen et al. [7] concluded that mental health levels declined during the pandemic, and this effect was strongest for women, and younger age groups. However, most COVID-19 research focused on health problems from infection or psychiatric conditions during the pandemic. Evidence on the possible impact of the pandemic situation on symptom reports has been scarce, or focus on a very narrow selection of symptoms. This study addresses this gap by examining perceived pandemic impacts on health symptoms, and relating findings to prior disaster research.

The aim of this study is to assess the association between perceived impact of (measures regarding) the COVID-19 pandemic and prevalence, duration and severity of health symptoms. At this point we would like to emphasize that this study does not focus on symptoms *during an infection* with COVID-19, but rather during the *period in time* of the COVID-19 pandemic. Two research questions were addressed:

1. What is the association between perceived impact of the pandemic and the reporting of health symptoms during the period of the pandemic?
2. Do people that got infected with COVID-19 experience more and longer lasting-symptoms?

## Methods

### Sample selection

At least once every four years, Community Health Services in the Netherlands (GGDs) are commissioned to monitor the health, well-being and lifestyle of adults and the elderly. This study used data from the GGD Health Monitor 2020: a collaboration between the 25 GGDs, the public health department of the municipality of Utrecht, the National Institute for Public health and the Environment (RIVM), and Statistics Netherlands (CBS).

Two separate samples within private households were selected: one for adults aged 18 to 64 years, one for elderly aged 65 years and older. A total of 68,383 adults and 39,339 elderly were invited to participate in the study. On the 29th of September 2020, an invitation letter for the digital survey was sent out. Four weeks later all non-respondents received a reminder that included both a link to the digital survey, and a printed version of the survey. After three more weeks, the non-respondents received a second reminder. 67% of the responding adults and 49% of the responding elderly responded digitally, all others filled out the paper version of the survey.

Participation in the GGD Health Monitor was voluntary. Completing the questionnaire, either online or on paper, indicated consent to participate. Several measurements were taken to handle all survey data confidentially, and to ensure that potential identifiability of individuals was minimized [10]. The legal grounds for GGD regio Utrecht and RIVM to process sensitive individual information is provided by Article 9(2)(i) of the General Data Protection Regulation (GDPR) [11], which permits the processing of such data when necessary for reasons of public interest in the area of public health.

### Measures

#### Health symptoms

To assess self-reported symptoms, The “Symptoms and Perceptions” (SaP) questionnaire [12] was used to assess self-reported symptoms. This questionnaire comprises 28 health symptoms in different organ systems, such as headache, fatigue, concentration problems, abdominal pain and sleep problems (see also Appendix A). Respondents indicated whether they experienced these symptoms in the past month (binary scale). If yes, they reported the duration of these symptoms (a 4-point subscale, with “over 6 months” being the highest value). The third subscale assessed whether participants consulted a general practitioner for their symptoms. A higher cumulative score in each of these subscales indicates increased symptom reporting, longer symptom duration, and higher (perceived) severity of symptoms, respectively. The SaP questionnaire has shown to have high reliability and good convergent validity [12]. The questions of the SaP-questionnaire are presented in Appendix A. Two additional items relevant to the symptomatic profile of COVID-19 as well as post-COVID conditions were added; throat symptoms, and problems with smell or taste. Internal consistency based on the data of the present study was also high (Cronbach’s a = 0.85).

#### Impact of the COVID-19 pandemic

To measure the perceived impact of the COVID-19 pandemic, respondents compared their situation at the timepoint of the survey (autumn 2020) to the pre-pandemic situation (February 2020). We used six dichotomous variables that indicated respondents respectively (1) feeling anxious, (2) feeling depressed, (3) feeling lonely, (4) being stressed, (5) a changed financial situation, and (6) experienced delayed care due to the COVID-19 pandemic.

#### COVID-19 infection

As large-scale COVID-19 testing was unavailable in the Netherlands during the survey (October/November 2020), respondents self-reported (suspected) infections, confirmed by a test or not. Anyone answering they got infected, whether or not confirmed by a test, was considered as a COVID-19-infected case in this study.

#### Demographic characteristics, lifestyle, chronic diseases and socio-economic status

Multiple variables were included as potential confounders: age (continuous), gender (dichotomous), household income (quintiles), educational level (categorical), migration background (categorical), obesity (BMI ≥ 30) (dichotomous), excessive use of alcohol (men > 14 glasses/week, women > 7 glasses/week) (dichotomous), smoking behavior (categorical), and longer lasting health symptoms (dichotomous). Educational level was measured in three categories: low, middle, and high educational level. Low are the ones that only completed primary education. The middle educated are the ones that completed prevocational secondary education (VMBO), senior general secondary education (HAVO), pre-university education (VWO), senior secondary vocational education (MBO). The higher educated are the ones with higher vocational education (HBO) and university education (WO).

### Statistical analyses

For the main analyses, negative binomial regression analyses were carried out, considering that the dependent variables (symptom scores) were count variables, and also to account for overdispersion. For each regression model, the dependent variable is mentioned on top of the columns. The main analyses concerned the association between perceived impact of the COVID-19 pandemic and symptom report, based on the total sample. All analyses were corrected for age (categories), gender (dichotomous), household income (quintiles), educational level (categories), migrational background (categories), obesity (dichotomous), excessive use of alcohol (dichotomous), smoking behaviour (categorical), chronic diseases (dichotomous), and COVID-19-status (dichotomous). There was no evidence for multicollinearity. When data were missing for a given variable, those cases were omitted from the analysis.

Given the overrepresentation of elderly individuals in our study, as sensitivity analyses, we repeated the analyses focusing solely on those aged 65 years and older. To enhance readability, only gender, age, COVID-19 infection, and pandemic-related aspects are presented in the main tables and text. The results for lifestyle factors are discussed in the main text of the results section, but are not presented in the tables. These results for lifestyle factors can be found in Appendix B, Tables B.1 and B.2. For each association incidence rate ratios (IRR), and 99% confidence intervals (CI) were calculated. We have employed a more conservative significance level (*p* < 0.01) in order to correct for multiple testing. Analyses were performed with STATA version 15.0 (StataCorp LP, College Station, TX, USA).

## Results

### Descriptive results and non-response

Response rate was 34% for the adults (*n* = 22,983) and 58% for the elderly (*n* = 22,920), representing 5.75% of the target population (province of Utrecht minus the city of Utrecht) (numbers retrieved from StatLine [13]). Non-response analysis showed respondents were somewhat older than the general population in Utrecht [13], predominantly of Dutch ethnicity (89% in sample versus 76% in the general Utrecht population), and had higher education and income levels. Non-western migration backgrounds (4% in sample versus 14% in general Utrecht population) and smokers (13% vs. 20%) were underrepresented. Table 1 shows the demographic characteristics of the full sample. The most reported symptoms in the past month were fatigue, back problems, neck and shoulder problems, muscle pain, and leg/hip/knee/foot problems (Table 2).

**Table 1:** Demographic characteristics

	*N*	Mean	Std. Dev	
Age	45,903	58.14	19.21	
Age 18–44	12,040	30.73	7.83	
Age 45–64	11,164	55.42	5.68	
Age 65–74	13,522	69.52	2.87	
Age 75 and older	9,177	80.65	4.72	

	N	Percentage		
Gender	45,903			
Male	22,334	48.65		
Female	23,569	51.35		
Household income in quintiles	45,633			
Quintile 1		7.19		
Quintile 2		15.17		
Quintile 3		19.58		
Quintile 4		25.05		
Quintile 5		33.01		
Educational level	43,640			
Lower		29.27		
Middle		30.65		
Higher		40.08		
Migrational background	45,903			
Dutch		88.86		
Western migration background		7.41		
Non-Western migration background		3.73		
Obesity (BMI ≥ 30)		13.05		
Excessive use of alcohol	7,571	17.41		
Smoking behavior	43,639			
Non-smokers		46.66		
Smokers		12.17		
Ex-smokers		41.17		
Long-lasting health conditions	15,865	35.92		
COVID-19 status	44,242			
Non-COVID-19 infected	41,484	93.77		
COVID-19 infected	2,758	6.23		

**Table 2:** Prevalence of health symptoms during the past month

Organ system	Symptom	Percentage
General and Unspecified	Fatigue	46
Digestive	Stomach	19
	Diarrhea	18
	Nausea	9
Eye	Eye	16
Ear	Ear	13
Cardiovascular	Palpitations	12
	Pain or pressure chest	8
Musculoskeletal	Back	36
	Neck and shoulder	36
	Muscle pain	34
	Leg/hip/knee/foot	34
	Arm/elbow/hand/wrist	22
Neurological	Headache	33
	Dizziness	20
	Tingling fingers/feet/toes	16
	Sensitivity to lights or sounds	7
	Loss of taste or smell	5
Psychological	Sleeping	27
	Irritable/angry	22
	Anxious feeling	21
	Memory or concentration	20
	Depressed feeling	14
	Acute stress/crisis	10
Respiratory	Nose	22
	Coughing	16
	Sore throat	10
	Shortness of breath	8
Skin	Skin	19
Endocrine/Metabolic and Nutritional	Weight change	10

### Association between perceived impact of the pandemic and symptom reporting

Table 3 shows the associations between pandemic impact and symptom reporting, adjusting for demographic characteristics. Analyses yielded consistent and significant associations between anxiety, depression, loneliness, stress, and delayed care due to the COVID-19 pandemic, as well as more longer-lasting and more severe symptoms. Financial impact showed no significant associations with symptom report. COVID-19 infection was also associated with increased symptom number, duration and severity. Women and individuals with pre-existing health issues were at greater risk for more frequent, longer lasting, and severe symptoms (Appendix B, Table B.1.). The association between delayed care and the number of symptoms and symptom duration declined when lifestyle factors were included, as did differences by education and income.

**Table 3:** Associations (IRR, 99% CI) between demographics and the impact of COVID-19 pandemic on number, duration, and severity of symptoms, based on the total sample (statistically significant results in **bold**)*

	Number of Symptoms				Duration of Symptoms				Severity of Symptoms			
	IRR	CI			IRR	CI			IRR	CI		
Age	**1.00**	**(1.00**	**-**	**1.00)**	**1.00**	**(1.00**	**-**	**1.00)**	**1.01**	**(1.01**	**-**	**1.01)**
Female	**1.28**	**(1.25**	**-**	**1.31)**	**1.30**	**(1.27**	**-**	**1.34)**	**1.25**	**(1.19**	**-**	**1.31)**
COVID patients	**1.28**	**(1.23**	**-**	**1.33)**	**1.21**	**(1.16**	**-**	**1.27)**	**1.24**	**(1.12**	**-**	**1.36)**
Impact of COVID Pandemic												
Anxiety	**1.21**	**(1.18**	**-**	**1.25)**	**1.22**	**(1.17**	**-**	**1.26)**	**1.29**	**(1.20**	**-**	**1.38)**
Depression	**1.25**	**(1.21**	**-**	**1.29)**	**1.27**	**(1.22**	**-**	**1.32)**	**1.29**	**(1.19**	**-**	**1.40)**
Loneliness	**1.19**	**(1.16**	**-**	**1.23)**	**1.19**	**(1.15**	**-**	**1.23)**	**1.17**	**(1.10**	**-**	**1.25)**
Stress	**1.27**	**(1.23**	**-**	**1.31)**	**1.22**	**(1.18**	**-**	**1.26)**	**1.22**	**(1.14**	**-**	**1.30)**
Financial	1.02	(0.99	-	1.06)	1.03	(0.99	-	1.07)	1.03	(0.96	-	1.11)
Delayed care	**1.26**	**(1.10**	**-**	**1.43)**	**1.51**	**(1.31**	**-**	**1.74)**	**1.63**	**(1.20**	**-**	**2.22)**

*Corrected for lifestyle factors and presence of chronic diseases, full results are shown in Appendix B, Table B.1

### People with and without a COVID-19 infection

COVID-19-infected individuals reported a higher prevalence of all symptoms (results not shown), with fatigue, nausea, chest pain or pressure, shortness of breath, coughing, sore throat, and loss of taste and smell at least twice as common compared to non-infected individuals. The largest difference was observed for loss of taste or smell (27.7% in infected vs. 5.1% in non-infected). Among those aged 65 years and older, the relative differences between infected and non-infected remained similar, although the prevalence of nasal symptoms among infected was no longer twice as high, and the fivefold prevalence of loss of taste or smell declined (9.7% in infected vs. 2.7% in non-infected).

### Population subgroups

#### Elderly

Delayed care due to the COVID-19 pandemic had greater impact on the elderly (Table 4). Symptom differences by income were weaker, with only the two lowest quintiles significantly differing from the highest quintile (except for quintile 4 for the number of symptoms). As in the full sample, those with longer during health problems reported more frequent, longer lasting, and severe symptoms than those without such longer during health symptoms (Appendix B, Table B.2.).

**Table 4:** Associations (IRR, 99% CI) between demographics and the impact of COVID-19 pandemic on number, duration, and severity of symptoms, based on people aged 65 years and older (statistically significant results in **bold**)*

	Number of symptoms				Duration of symptoms				Severity of symptoms			
	IRR	CI			IRR	CI			IRR	CI		
Age	**1.01**	**(1.00**	**-**	**1.01)**	**1.01**	**(1.01**	**-**	**1.02)**	**1.02**	**(1.02**	**-**	**1.03)**
Female	**1.27**	**(1.22**	**-**	**1.32)**	**1.25**	**(1.20**	**-**	**1.30)**	**1.14**	**(1.07**	**-**	**1.22)**
COVID patients	**1.26**	**(1.15**	**-**	**1.39)**	**1.20**	**(1.08**	**-**	**1.33)**	1.17	(0.96	**-**	1.43)
Impact of COVID Pandemic												
Anxiety	**1.22**	**(1.16**	**-**	**1.28)**	**1.15**	**(1.09**	**-**	**1.23)**	**1.17**	**(1.06**	**-**	**1.29)**
Depression	**1.29**	**(1.21**	**-**	**1.38)**	**1.26**	**(1.17**	**-**	**1.36)**	**1.29**	**(1.15**	**-**	**1.46)**
Loneliness	**1.21**	**(1.15**	**-**	**1.27)**	**1.21**	**(1.14**	**-**	**1.28)**	**1.19**	**(1.09**	**-**	**1.30)**
Stress	**1.37**	**(1.29**	**-**	**1.46)**	**1.29**	**(1.21**	**-**	**1.38)**	**1.27**	**(1.14**	**-**	**1.42)**
Financial	1.07	(0.98	-	1.16)	1.03	(0.95	-	1.13)	1.06	(0.93	-	1.21)
Delayed care	**1.62**	**(1.24**	**-**	**2.13)**	**1.82**	**(1.31**	**-**	**2.55)**	1.45	(0.78	**-**	2.72)

*Corrected for lifestyle factors and presence of chronic diseases, full results are shown in Appendix B, Table B.2

## Discussion

This study investigated non-specific health symptoms reported by adults and elderly in Utrecht, the Netherlands, during the COVID-19 pandemic. Findings showed that pandemic-related anxiety, depression, loneliness, stress, and delayed care were associated with more, longer lasting, and more severe symptoms. Financial impact of the pandemic was not significantly associated with symptoms. To our knowledge, this is one of the first studies to link perceived pandemic impact to number, duration, and severity of non-specific health symptoms.

In addition, people infected with COVID-19-infected individuals reported worse symptom outcomes. A possible explanation is that (atypical) symptoms of COVID-19 overlap with the symptoms included in the Symptoms and Perceptions questionnaire [12, 14]. Women and individuals with co-morbidities also experienced more severe and persistent symptoms, aligning with broader health literature [15–17]. Furthermore, subgroup analyses showed delayed care impacted the elderly the most.

Previous studies on COVID-19 and health focused on health problems due to the infection, or psychological conditions during the pandemic, but not on the types of (acute) health symptoms that most people experience. This study supports previous findings that the COVID-19 pandemic had negative impact on physical and mental health (e.g [7, 18]). To limit spread of the virus, many countries implemented various COVID-19 measures, such as stay at home requirements, closing of workplaces and school, and restrictions on international and domestic travel. These measurements likely triggered or contributed to (increased) feelings of loneliness, stress, anxiety, and depression. This study did not include questions on *why* people experienced the symptoms they reported; we do not know for sure this was due to the measurements at that point in time. Other studies, however, have shown that the measurements impacted physical and mental health [6]. The perceived impact of a pandemic is a possible determinant of health symptoms. A better insight into symptom report and associated risk factors could help public health professionals and policy makers to identify symptom profiles, vulnerable groups and improve aftercare, during and in the aftermath of a pandemic. Further research on this specific topic is recommended. At a later time, the pandemic would become more intense in terms of number of infections and measures. It is feasible that there is substantial overlap between long-covid profiles and the dominant profile of patients with non-specific symptoms. An updated study could for example look into the changes over time, and whether symptom profiles differed as the COVID-19 persisted.

This study was conducted between the first and second wave of COVID-19. There were various measures at play, and there was a lot of fear and insecurity, and it is interesting to see how this is reflected in more objective data. We compared prevalence of symptoms in our study to symptom prevalence in a nation-wide general practitioner (GP) database from the Netherlands Institute for Health Services Research (Nivel) [19, 20]. The period in time comparable to the period at which the survey was held, is week 36 to 44 (September/October 2020). Due to the COVID pandemic, a relatively small share of people visited the GP with their health symptoms. Interestingly, for nausea and muscle pain, for this period in 2020 the highest peak in prevalence in the past three years was found. This might be an indication that nausea and muscle pain are associated with (the aftermath of) a COVID-19 infection.

Future research should prioritize longitudinal designs to investigate symptom report during and in the aftermath of the pandemic. Such approaches would provide more insight into the detailed symptom profiles and progression of symptoms over time. This can help policy makers and healthcare professionals develop more effective strategies to improve care during and after pandemics, with a specific focus on addressing the needs of vulnerable groups. Additionally, future studies should preferably adjust for variables such as age, gender, household income, obesity, and lifestyle aspects such as smoking behaviour, as these were identified as significant determinants of health symptom report. Of all pandemic aspects, delayed care was the most strongly associated with health symptoms. This especially affected elderly. This underscores the need for tailored healthcare delivery models in times of pandemics, to ensure continuity of care.

### Strengths and limitations

This study provided insight in health symptoms during the period of the COVID-19 pandemic. One of the major strengths of this study, is the large study population of almost a full Dutch province. Validated instruments were used to assess health symptoms and individual characteristics. The downside of a large sample size can be that analyses are overpowered. Effect sizes are relatively small, and therefore the associations found should be interpreted carefully. A study limitation is the cross-sectional study design that does not allow inferences about causality. Also, reliance on self-reported data may be accompanied by reporting biases. Since perceived impact of the COVID-19 pandemic is asked in retrospect, memory bias could be present. COVID-19 status is also measured by self-reports. In addition, it is possible that the number of COVID-19-cases is an overestimation of actual COVID-19 cases at the time. Despite this not being a focus of the study, it is important to keep in mind when interpreting differences between COVID-19 and non-COVID-19 respondents. It could be the case that those with little to no symptomatic effects from a COVID-19 infection reported that they have not been infected, or those with a regular cold might report that they were infected with COVID-19. In addition, some population groups were over- or underrepresented in the current sample, such as elderly, people with Dutch ethnicity, people with a non-western migration background, higher educated, and higher income groups. and higher educated.

## Conclusion

Pandemic stressors such as anxiety, depression, loneliness, stress and delayed cared were consistently associated with an increase in number, duration and severity of symptoms, suggesting that perceived impact of a pandemic could be a possible determinant of experiencing health symptoms, independently of health status and socio-demographic characteristics. The present study also found association between having or suspecting a COVID-19 infection and symptom number, perceived severity, and duration. In the light of post-COVID-19 conditions, this highlights the need for early targeted interventions to address health impacts and identify vulnerable groups during and after pandemics, to improve aftercare during and in the aftermath of pandemics.

## Appendix A


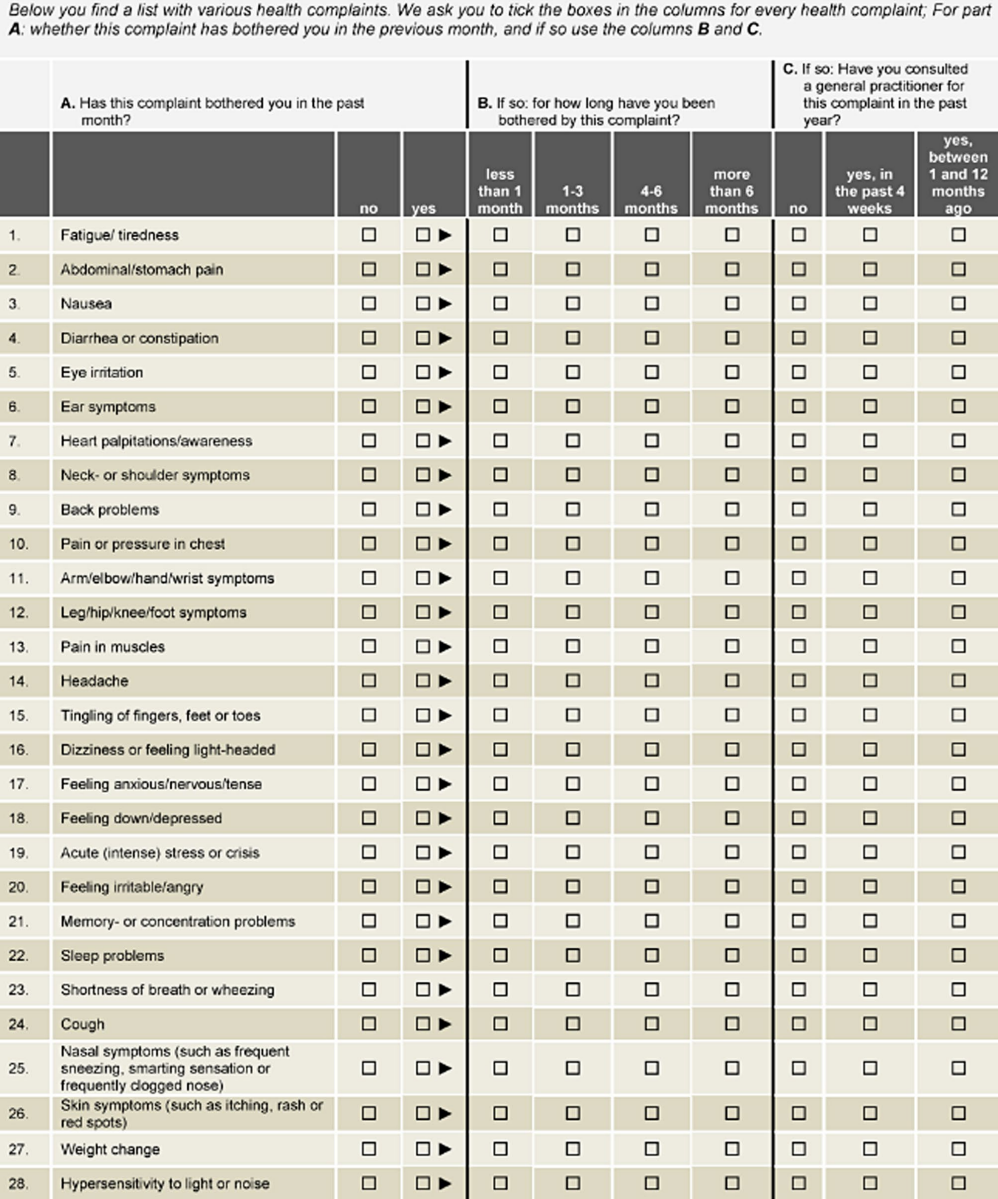


The SaP-questionnaire [11]. For the health monitor that provided data for this currect article, two extra symptoms were added: throat symptoms, and problems with smell or taste.

## Appendix B

**Table B.1:** Associations (IRR, 99% CI) between demographics, impact of COVID-19 pandemic, and lifestyle factors on number, duration, and severity of symptoms, based on the total sample (statistically significant results in **bold**)*

	Number of symptoms		Duration of symptoms		Severity of symptoms	
	IRR	CI	IRR	CI	IRR	CI
Age	**1.00**	**(1.00-1.00)**	**1.00**	**(1.00-1.00)**	**1.01**	**(1.01-1.01)**
Female	**1.28**	**(1.25-1.31)**	**1.30**	**(1.27-1.34)**	**1.25**	**(1.19-1.31)**
COVID infected	**1.28**	**(1.23-1.33)**	**1.21**	**(1.16-1.27)**	**1.24**	**(1.12-1.36)**
Impact of COVID pandemic						
Financial	1.02	(0.99-1.06)	1.03	(0.99-1.07)	1.03	(0.96-1.11)
Anxiety	**1.21**	**(1.18-1.25)**	**1.22**	**(1.17-1.26)**	**1.29**	**(1.20-1.38)**
Depression	**1.25**	**(1.21-1.29)**	**1.27**	**(1.22-1.32)**	**1.29**	**(1.19-1.40)**
Loneliness	**1.19**	**(1.16-1.23)**	**1.19**	**(1.15-1.23)**	**1.17**	**(1.10-1.25)**
Stress	**1.27**	**(1.23-1.31)**	**1.22**	**(1.18-1.26)**	**1.22**	**(1.14-1.30)**
Delayed care	**1.26**	**(1.10-1.43)**	**1.51**	**(1.31-1.74)**	**1.63**	**(1.20-2.22)**
Household income (Quintile 5 = reference category)						
Quintile 1	**1.13**	**(1.08-1.19)**	**1.22**	**(1.16-1.29)**	**1.28**	**(1.16-1.42)**
Quintile 2	**1.13**	**(1.09-1.18)**	**1.16**	**(1.11-1.21)**	**1.29**	**(1.20-1.40)**
Quintile 3	**1.07**	**(1.03-1.10)**	**1.09**	**(1.05-1.13)**	**1.15**	**(1.08-1.23)**
Quintile 4	**1.05**	**(1.02-1.08)**	**1.05**	**(1.02-1.08)**	**1.10**	**(1.03-1.16)**
Educational level (higher education = reference category)						
Lower education	0.97	(0.94-1.00)	**1.07**	**(1.03-1.11)**	**1.31**	**(1.23-1.40)**
Middle education	**1.03**	**(1.01-1.06)**	**1.08**	**(1.05-1.11)**	**1.25**	**(1.19-1.32)**
Migrational background (autochthonous = reference category						
Western	**1.07**	**(1.03-1.12)**	**1.09**	**(1.03-1.14)**	1.06	(0.97-1.16)
Non-Western	1.00	(0.94-1.07)	1.07	(1.00-1.16)	**1.39**	**(1.23-1.57)**
Obesity	**1.12**	1.16)**(1.08-**	**1.13**	**(1.09-1.17)**	**1.20**	**(1.12-1.27)**
Excessive use of alcohol	1.01	(0.98-1.04)	1.00	(0.97-1.03)	**0.93**	**(0.88-0.98)**
Smoking behavior (non-smokers = reference category)						
Smoker	**1.13**	**(1.09-1.17)**	**1.17**	**(1.12-1.22)**	**1.24**	**(1.15-1.34)**
Ex-smoker	**1.10**	**(1.07-1.12)**	**1.09**	**(1.06-1.12)**	**1.12**	**(1.06-1.18)**
Longer during health problems	**1.65**	**(1.61-1.68)**	**1.83**	**(1.79-1.88)**	**2.25**	**(2.15-2.36)**

**Table B.2:** Associations (IRR, 99% CI) between demographics, impact of COVID-19 pandemic, and lifestyle factors on number, duration, and severity of symptoms, based on people aged 65 years and older (statistically significant results in **bold**)*

	Number of symptoms		Duration of symptoms		Severity of symptoms	
	IRR	CI	IRR	CI	IRR	CI
Age	**1.01**	**(1.00-1.01)**	**1.01**	**(1.01-1.02)**	**1.02**	**(1.02-1.03)**
Female	**1.27**	**(1.22-1.32)**	**1.25**	**(1.20-1.30)**	**1.14**	**(1.07-1.22)**
COVID infected	**1.26**	**(1.15-1.39)**	**1.20**	**(1.08-1.33)**	1.17	(0.96-1.43)
Impact of COVID Pandemic						
Financial	1.07	(0.98-1.16)	1.03	(0.95-1.13)	1.06	(0.93-1.21)
Anxiety	**1.22**	**(1.16-1.28)**	**1.15**	**(1.09-1.23)**	**1.17**	**(1.06-1.29)**
Depression	**1.29**	**(1.21-1.38)**	**1.26**	**(1.17-1.36)**	**1.29**	**(1.15-1.46)**
Loneliness	**1.21**	**(1.15-1.27)**	**1.21**	**(1.14-1.28)**	**1.19**	**(1.09-1.30)**
Stress	**1.37**	**(1.29-1.46)**	**1.29**	**(1.21-1.38)**	**1.27**	**(1.14-1.42)**
Delayed care	**1.62**	**(1.24-2.13)**	**1.82**	**(1.31-2.55)**	1.45	(0.78-2.72)
Household income (Quintile 5 = reference category)						
Quintile 1	**1.14**	**(1.03-1.25)**	**1.20**	**(1.09-1.33)**	**1.19**	**(1.03-1.38)**
Quintile 2	**1.11**	**(1.04-1.17)**	**1.13**	**(1.05-1.20)**	**1.23**	**(1.12-1.36)**
Quintile 3	1.04	(0.99-1.10)	1.05	(0.99-1.12)	1.13	(1.03-1.23)
Quintile 4	**1.05**	**(1.00-1.11)**	1.04	(0.98-1.09)	1.06	(0.97-1.15)
Educational level (higher education = reference category)						
Lower education	0.97	(0.93-1.02)	1.05	(1.00-1.11)	**1.22**	**(1.13-1.32)**
Middle education	1.03	(0.98-1.08)	1.05	(1.00-1.11)	**1.21**	**(1.11-1.31)**
Migrational background (autochthonous = reference category						
Western	1.05	(0.98-1.12)	**1.08**	**(1.01-1.16)**	1.02	(0.91-1.15)
Non-Western	1.05	(0.90-1.22)	1.06	(0.89-1.25)	**1.51**	**(1.16-1.95)**
Obesity	**1.13**	**(1.07-1.18)**	**1.14**	**(1.08-1.21)**	**1.15**	**(1.06-1.25)**
Excessive use of alcohol	1.03	(0.98-1.08)	1.01	(0.96-1.06)	0.96	(0.89-1.03)
Smoking behavior (non-smokers = reference category)						
Smoker	1.04	(0.97-1.12)	1.06	(0.98-1.14)	1.00	(0.89-1.13)
Ex-smoker	**1.09**	**(1.04-1.14)**	**1.06**	**(1.01-1.11)**	1.04	(0.97-1.11)
Longer during health problems	**1.83**	**(1.76-1.90)**	**1.89**	**(1.82-1.97)**	**2.01**	**(1.88-2.13)**

## Data Availability

No datasets were generated or analysed during the current study.

## Abbreviations

IRR: Incidence rate Ratios
NSS: Non-specific symptoms
GGD: Community health services
RIVM: National institute for public health and the environment
CBS: Netherlands Statistics
GDPR: General data protection regulation
SaP: Symptoms and Perceptions
CI: Confidence Intervals
